# Humoral and Cellular Immunity After Vaccination Against SARS-CoV-2 in Relapsing-Remitting Multiple Sclerosis Patients Treated with Interferon Beta and Dimethyl Fumarate

**DOI:** 10.3390/biomedicines13010153

**Published:** 2025-01-09

**Authors:** Marcin Bazylewicz, Monika Zajkowska, Monika Gudowska-Sawczuk, Rafał Kułakowski, Jan Mroczko, Dagmara Mirowska-Guzel, Joanna Kulikowska-Łoś, Agata Czarnowska, Barbara Mroczko, Jan Kochanowicz, Alina Kułakowska

**Affiliations:** 1 Department of Neurology, Medical University of Bialystok, 15-276 Bialystok, Poland; joannaakulikowska@gmail.com (J.K.-Ł.); zajkowskaagata@gmail.com (A.C.); jan.kochanowicz@uskwb.pl (J.K.); alakul@umb.edu.pl (A.K.); 2 Department of Neurodegeneration Diagnostics, Medical University of Bialystok, 15-269 Bialystok, Poland; monika.zajkowska@umb.edu.pl (M.Z.); mjanek2003@gmail.com (J.M.); barbara.mroczko@umb.edu.pl (B.M.); 3 Department of Biochemical Diagnostics, Medical University of Bialystok, 15-269 Bialystok, Poland; monika.gudowska-sawczuk@umb.edu.pl; 4 Department of Clinical and Experimental Pharmacology, Faculty of Medicine, Medical University of Warsaw, 02-091 Warsaw, Poland; rafal.kulakowski@icloud.com (R.K.); dagmara.mirowska-guzel@wum.edu.pl (D.M.-G.)

**Keywords:** COVID-19, SARS-CoV-2, vaccine, multiple sclerosis, humoral, cellular, immunity

## Abstract

Background/Objectives: The impact of vaccines against SARS-CoV-2 on the immunity of patients with multiple sclerosis (PwMS) is still not fully known. Further clarification could help address medical concerns related to the use of immunosuppressive and immunomodulatory medications, known as disease-modifying therapies (DMTs), in PwMS, as well as ensure adequate protection against severe outcomes of COVID-19. Therefore, the aim of our study was to evaluate the humoral and cellular immune response in PwMS treated with DMTs. Methods: The concentrations of IgG Spike (S) anti-SARS-CoV-2 antibodies and IgG Nucleocapsid (N) anti-SARS-CoV-2 antibodies, as well as interferon-gamma *(IFN-*γ) titers were analyzed in PwMS groups treated with dimethyl fumarate (DMF), interferon beta (IFN), and healthy control group. Results: Almost 100% of PwMS experienced seroconversion, which resulted from either vaccination and/or prior infection. Additionally, there were no significant differences between the study and control groups in terms of IgG (S) and (N) anti-SARS-CoV-2 antibody levels. However, interferon-gamma titers were lower in both PwMS groups, which may indicate adequate humoral and decreased cellular response to the examined PwMS. Additionally, after the division of the whole study group into two subgroups according to the time since the last vaccination, IgG (S) anti-SARS-CoV-2 and *IFN-*γ concentrations were significantly lower in the case of patients who were immunized more than 200 days before sample collection. No differences were observed in the case of subgroups in which sample collection was less than 200 days after vaccination when compared to the control group. Conclusions: This could indicate a time-related decrease in immunity in PwMS treated with DMTs.

## 1. Introduction

In 2019, the severe respiratory syndrome coronavirus 2 (SARS-CoV-2) was found in Wuhan, China. Infection with this virus causes a disease that affects the respiratory tract system, known as COVID-19. It spread worldwide, and on 11 March 2020, when nearly 118,000 confirmed cases were detected, the World Health Organization (WHO) announced COVID-19 as a pandemic [1,2]. Until 13 April 2024, almost 705 million confirmed cases had been reported, resulting in almost 7 million deaths [3]. The pandemic status continued until 5 May 2023 [4]. COVID-19 has a variety of clinical courses, ranging from asymptomatic, through symptoms like fatigue, fever, diarrhea, cough, dyspnea, malaise, myalgia, rhinitis, headache, diarrhea, chest pain [5], to severe outcomes such as acute respiratory distress syndrome (ARDS) or even death [6]. The majority of clinical cases result in asymptomatic or mild outcomes [5]. COVID-19 infection initiates when the spike protein of SARS-CoV-2, one of the viruses four structural proteins (spike, nucleocapsid, envelope, and membrane), binds to receptors on the host cell surface (angiotensin-converting enzyme 2 [ACE2] or Neuropilin-1 [NRP-1] receptors) [7,8]. The SARS-CoV-2 (N) protein, which is connected with viral RNA, seems to be responsible for RNA transcription and replication in host cells. The reverse transcription polymerase chain reaction tests (PCR-RT), as well as antigen or serological tests, play an important role in diagnostics procedures used for COVID-19 diagnosis [9].

PCR-RT and antigen methods are useful for confirming the ongoing SARS-CoV-2 infection [10]. The serological method detects antibodies produced when a patient is exposed to the virus. This immune response is known as the humoral response [11]. In 2020, the first vaccines against COVID-19 were introduced. Various vaccine platforms were developed, including mRNA vaccines, where mRNA coding spike protein is implemented (BNT162b2, mRNA-1273) [12]; protein subunit vaccines, where spike protein is used for immunization (NVX-CoV2373) [13]; and vector vaccines, which involve prepared adenoviruses transferring genetic material to host cells (ChAdOx1 nCoV-19, Ad26.COV2.S) [14]. Two types of immunization can be distinguished: active immunization, in which the patient is exposed to SARS-CoV-2 infection, and passive immunization, which occurs through vaccination. Despite the presence of five classes of antibodies, only three of them (IgM, IgA, and IgG) are relevant for determining the type of immunity in patients [11]. The presence of antibodies in IgM and IgA classes detected in human serum or plasma is present early, about 6–8 days after infection [15], and after 49–56 days after immunization onset, they become negative. IgG class antibodies can be detected 9–13 days after infection and may last up to 80–105 days or even longer [16,17,18]. Anti-SARS-CoV-2 N antibodies play a significant role in humoral immunity obtained after infection. However, those antibodies are absent in humoral immunity reached after vaccination, where anti-SARS-CoV-2 S antibodies are present [19]. Additionally, after immunization, SARS-CoV-2 antigens activate naive lymphocytes T, causing them to release a substantial amount of interferon-gamma. What is more, after immunization, naive lymphocytes T are activated, resulting in the release of a substantial amount of interferon-gamma. Some studies indicate that cellular immunity lasts longer than humoral, even for years [20,21,22]. The fact of obtaining humoral and cellular immunity may be linked with a lower risk of severe infection outcomes [23].

Multiple sclerosis (MS) is a chronic autoimmune demyelinating disease that affects 2.8 million people worldwide [24,25] and almost 45,000 individuals in Poland [25]. A typical patient diagnosed with MS is an approximately 32-year-old female. Many patients with multiple sclerosis (PwMS) are treated with disease-modifying treatments (DMTs), which are immunomodulating and immunosuppressive medications aimed at reducing progressive disability and radiological progression of the disease [26,27]. This approach is referred to as NEDA 3 (no evidence of disease activity), indicating the absence of clinical and radiological signs of disease activity [28]. Those patients with autoimmune disease and specific treatment applied constitute a group with unclear immunity status.

It remains unknown what their response was after vaccination against SARS-CoV-2. Available scientific reports on the influence of Disease-Modifying Therapies (DMTs) on humoral and cellular immunity against SARS-CoV-2 primarily focus on medications used in High Efficacy Treatments (HETs), such as natalizumab, fingolimod, alemtuzumab, ocrelizumab, and cladribine [29]. Data regarding other drugs, such as interferon beta (IFN) and dimethyl fumarate (DMF) or glatiramer acetate, remains limited, particularly for those who received their last dose of SARS-CoV-2 vaccination more than 200 days prior. In the available literature, immunomodulatory therapies such as DMF (dimethyl fumarate) and INF (interferon) are mostly described as safe due to their mechanisms of action. DMF induces the depletion of memory T cells, reduces the count of activated T cells, and supports the expansion of naïve T cells [30]. However, some scientists have reported that people with multiple sclerosis (PwMS) treated with DMF could experience low levels of lymphocytes in peripheral blood, which may decrease the development of immune responses [31]. Interferon beta, in general, suppresses inflammation by decreasing the activation of T cells and inducing the differentiation of oligodendrocytes. The direct mechanism of action of interferon-beta is still unknown [32]. It is important to note that interferon and dimethyl fumarate are commonly used as primary DMT choices [33,34]. Therefore, the aim of our study was to evaluate the humoral and cellular immune response in PwMS treated with dimethyl fumarate and interferon beta.

## 2. Materials and Methods

### 2.1. Patients

The intended PwMS group being treated with dimethyl fumarate (DMF) and interferon (INF) consisted of approximately 100 patients (65 and 35 individuals, respectively). This calculation was based on the number of patients receiving treatment with the selected drugs at the clinic. Some patients were excluded from the study due to exclusion criteria such as DMT treatment change detected in the survey, absence, refusal to sign the consent, incorrect survey completion, and lack of only qualitative results in the tests conducted (details available in Supplementary Figure S1). Finally, the study group consisted of 72 PwMS, who were treated with dimethyl fumarate (*n* = 48) or interferon beta (*n* = 24) in a national drug funding program conducted by the Department of Neurology, Medical University of Bialystok. Every case of MS was diagnosed using McDonald criteria (2017) [35] and strictly monitored in accordance with the Polish national drug program guidelines. All patients were treated with current DMT at least 12 months before the first dose of anti-SARS-CoV-2 vaccination and with the Expanded Disability Status Scale (EDSS) [36] below 4.5. 72.2% of PwMS studied in the investigation were women. The mean age was 40.4 years in the subgroup treated by DMF and 48.63 years in the subgroup treated by IFN. The managed control group, which was comparable to both study groups, consisted initially of 40 participants. Due to patients’ refusals during data collection and only qualitative results in the case of few patients, the control group ultimately comprised 28 physically healthy individuals without a diagnosis of MS. This group closely resembled the MS group in terms of age and sex and did not have any significant comorbidities; most participants were free from medical conditions, with only a small number reporting hypertension, diabetes managed with oral medication, or hypothyroidism (Table 1). All participants received at least two doses of the mRNA vaccine or at least one dose of Ad26.COV2.S.

### 2.2. Patients Vaccination Status

The mean time from the last vaccination against SARS-CoV-2 to blood sample collection was 320.56 days in the subgroup of PwMS treated with DMF, 320.5 days in the subgroup of PwMS treated with IFN, and 320.54 days in the whole study group as well as 316.29 in the control group (Table 2). The shortest interval between the last dose of vaccination against SARS-CoV-2 and sample collection was 84 days. In the whole study and control groups, the most frequent vaccine used in all doses was BNT162b2 (Table 3). The longest mean interval between the first and the second dose of vaccination was in PwMS treated with DMF 47.35 days (vs. INF 43.04 days vs. Control 40.43 days). Although mean intervals between the second and third doses of immunization against SARS-CoV-2 were evaluated, the control group had the longest mean—286.86 days (vs. INF 190.73 days; vs. DMF 224.61 days) and 312.95 days (vs. INF 231.6 days; vs. DMF 261.18 days), respectively.

The third dose of vaccination against SARS-CoV-2 was chosen more frequently by the individuals from the control group rather than the study group (Table 3).

### 2.3. Biochemical Analyses

Blood samples were collected during routine control visits with participants diagnosed with multiple sclerosis enrolled in a pharmacological treatment program spanning from 30 May 2022 to 16 March 2023. Analysis of humoral immunity against SARS-CoV-2 was performed by chemiluminescent microparticle immunoassay (CMIA). The concentrations of IgG antibodies against the receptor binding domain of S1 protein [AU/mL] and against n protein were assessed using the automatic Alinity system (Abbott, Abbott Park, Illinois, IL, USA). Results ≥ 50 AU/mL for IgG (S) anti-SARS-CoV-2 and ≥1.4 IgG (N) anti-SARS-CoV-2 were considered positive. The concentrations of interferon-gamma were determined using a Euroimmun Quan-T-Cell ELISA kit to assess the cellular immune response also in patients without detectable antibodies. Results ≥ 200 mIU/mL were marked as positive.

### 2.4. Surveys

Simultaneously to the blood samples collection, the data about the duration of the disease, DMT drug usage, details of vaccination against SARS-CoV-2 (date of vaccination, type of vaccine, possible adverse effects after vaccination, date of possible SARS-CoV-2 infection, neurological condition, date of last relapse) were collected from patients. To minimize the potential for errors during questionnaire completion, a physician was present to provide assistance when necessary.

### 2.5. Statistical Analysis

The data obtained from the questionnaires and investigations performed on blood samples were statistically analyzed. The analysis used statistical tests with a significance level of α = 0.05. The Shapiro-Wilk test was used to determine whether the variable distributions were normal. As all results were not normally distributed, the Kruskal–Wallis rank sum test was used to examine differences between all groups. Statistically significant differences were defined as comparisons resulting in *p*  <  0.05. All statistical analyses were performed using Statistica 13.1.

## 3. Results

### 3.1. Analysis of Humoral Immunity Against SARS-CoV-2

All of the examined groups presented nearly 100% positive results of IgG (S) anti-SARS-CoV-2 antibodies. Only two patients from the subgroup of PwMS treated with DMF did not reach the concentration of antibodies higher than 50 AU/mL in serum. The mean concentration of IgG (S) anti-SARS-CoV-2 antibodies in serum of PwMS treated with DMF was 15,506.18 AU/mL, treated with IFN was 22,450.1 AU/mL, and in the control group was 21,369.09 AU/mL. Performed statistical analysis revealed that there were no significant differences between concentrations of IgG (S) anti-SARS-CoV-2 antibodies (DMF vs. control group, *p* = 0.08; IFN vs. control group, *p* = 0.09; DMF + IFN vs. control group, *p* = 0.051). This may indicate that the humoral response of all tested groups was generally similar. Several studies found that after 6 months from vaccination, its effectiveness was evaluated as the concentration of IgG (S) antibodies against SARS-CoV-2 was much lower than after 7–60 days from immunization and that it continued to decrease at a different rate [37,38,39,40,41,42]. Many accessible articles separated patients into groups based on whether blood samples were acquired within 180 days after immunization against SARS-CoV-2. However, we chose a division at 200 days to achieve a more proportionate grouping in terms of patient numbers while ensuring that this approach did not significantly deviate from established trends in the literature and that the assessed results did not deviate significantly from those obtained when the cutoff at 180 days was established.

According to these facts, we have divided all tested groups into two subgroups: individuals whose blood samples have been taken less than 200 days and those whose blood has been drawn more than 200 days after the last dose of vaccination. Obtained mean values in all studied groups were as follows: 29,840.93 AU/mL in PwMS treated with DMF if blood collection was performed less than 200 days, and 8990.37 AU/mL if later than 200 days after vaccination; 61,966.05 AU/mL in subgroup of PwMS treated with IFN if blood collection was less than 200 days and 9278.12 AU/mL for patients treated with IFN if blood collection was more than 200 days since last dose of vaccination. The control group reached 32,029.68 AU/mL and 16,319.34 AU/mL, respectively. Obtained concentrations were significantly higher in the study subgroups, where the time from vaccination to sample collection was less than 200 days when compared to those where the time from vaccination to the collection of samples was more than 200 days (DMF, *p* = 0.0013; IFN, *p* = 0.0027; control group, *p* = 0.2281). The mean concentration of IgG (S) anti-SARS-CoV-2 antibodies was highest in PwMS subgroup treated by IFN, collected in less than 200 days after last dose of vaccine against SARS-CoV-2 (details were presented in Table 4), indicating no statistically significant differences (DMF vs. control, *p* = 0.7; IFN vs. control, *p* = 0.35; DMF + IFN vs. control, *p* = 0.95). After 200 days, IgG (S) anti-SARS-CoV-2 levels in study subgroups were lower than in the control group, which was statistically significant (DMF vs. control, *p* = 0.037; IFN vs. control, *p* = 0.015; DMF + IFN vs. control, *p* = 0.012). This confirms the previous assumptions about similar humoral responses in the first months after vaccination in the studied groups and their earlier decrease with time. Due to the fact that after division into subgroups, the number of patients in some cases was less than 20, this is a limitation of our study, and the results should be interpreted with caution. However, it is worth noting that both of the patient groups, as well as the control group, were strictly selected, which, on the other hand, may indicate a high reliability of the presented results.

Furthermore, the concentration of IgG (N) SARS-CoV-2 antibodies in all groups was measured. In subgroups of PwMS treated with DMF, IFN and in the control group, (50%, 66.67%, and 57.14%, respectively) were negative, which indicates that only part of the examined individuals had direct contact with the virus due to infection. Mean concentrations of IgG (N) anti-SARS-CoV-2 antibodies in serum PwMS treated with DMF and IFN in the whole study group and in the control group were as follows: 2.46, 2.17, 2.36, and 1.82, respectively. Details are described in Table 5. The acquired data were compared, and there were neither statistically significant differences between the overall study group vs. control group (*p* = 0.8478) nor between the separated subgroups vs. control group (DMF *p* = 0.58, IFN *p* = 0.63), which also confirms similar humoral response of all patients. Due to the fact that precise determination of the last SARS-CoV-2 infection is impossible, IgG (N) antibodies were only assessed as one group, without the additional subgroup division.

### 3.2. Analysis of Cellular Immunity Against SARS-CoV-2

As data presented so far revealed that the decrease in IgG (S) anti-SARS-CoV-2 concentration may be compensated by cellular immunity, which is characterized as interferon-gamma concentration [43], it was measured to assess the cellular response in tested groups. In the study subgroups, PwMS treated with DMF, PwMS treated with IFN, and in the control group, negative results were obtained (20.84%, 12.5%, and 3.57%, respectively). Mean concentrations of interferon-gamma in tested groups were respectively 1659.41 mIU/mL, 1613.81 mIU/mL, and 2904.46 mIU/mL. A statistical analysis was performed, and it revealed that the titers obtained by PwMS treated with DMF and IFN were lower than those obtained in the control group, which was statistically significant (*p* = 0.001). Interferon-gamma concentration in serum measured in both groups separately was also lower, although only in the case of groups treated with DMF, statistical significance has been proved (*p* = 0.0008). Obtained data draw attention to the fact that the quick and violent response of T-cells is a crucial factor in mild COVID-19 outcomes [44]. Taking the above into consideration, the decision to conduct a more detailed analysis of the obtained results was made.

Participants in the investigation were divided into two groups, as described before. One group took into consideration individuals whose time between the last vaccination against SARS-CoV-2 and the blood drawn was less than 200 days, and the second one where that period lasted more than 200 days. When considering <200 days groups, the highest mean of interferon-gamma concentration was obtained in the group of PwMS treated with interferon beta, although when we considered groups with >200 days period, the control group reached the highest concentration. The study compared single subgroups and the whole study group to the control group. When comparing the <200 days groups, mean differences were not statistically significant (DMF vs. control, *p* = 0.61; IFN vs. control, *p* = 0.2; DMF + IFN vs. control, *p* = 0.92). However, when the time between the last passive immunization by SARS-CoV-2 and the collection of a blood sample was more than 200 days, the highest mean was reached by the control group (3004.94 mIU/mL vs. 1374.61 mIU/mL (DMF) vs. 1520.30 mIU/mL (IFN), respectively), what was statistically significant (DMF vs. control, *p* = 0.00045; IFN vs. control, *p* = 0.004; DMF + IFN vs. control, *p* = 0.0002).

### 3.3. Questionnaire Analysis

Individuals in the completed survey study reported similar percentages of troubling symptoms in the time between their last SARS-CoV-2 immunization (DMF 22.92% vs. IFN 20.83%). Contact with the infected person was observed more frequently in the PwMS group treated with dimethyl fumarate than in other groups (83.33% for DMS vs. 66.67% for IFN vs. 35.71% for the control group).

Interestingly, patients treated with IFN beta were more likely to suspect that they were suffering from COVID-19 (IFN 50% vs. DMF 37.5% vs. control group 35.71%). What is more, when participants were asked about attendant symptoms, they provided interesting information. PwMS treated with DMF and IFN complained about the cough most frequently (27.08%; 33.33%, respectively), followed by fever (16.67%; 25%, respectively) and dyspnea (10.42%; 20.83%, respectively). Different results were obtained in the control group, where the most frequent symptoms were fever and cough (both 28.57%), followed by dyspnea 21.43%.

Additional data were also obtained regarding adverse occurrences after vaccination against SARS-CoV-2. In the study subgroup treated with dimethyl fumarate after the first dose of vaccination, the most frequent symptoms were local pain 37.5% (*n* = 18), fever and fatigue 16.67% (*n* = 8) as well as headache 10.42% (*n* = 5). Following the second dose of vaccination, the most prevalent were local pain 26.09% (*n* = 12), fever and fatigue 19.57% (*n* = 9), and muscle pain 17.39% (*n* = 8). A survey in the subgroup treated with IFN revealed that after the first dose of treatment, the most common adverse events were local pain 41.67% (*n* = 10), fever, and fatigue 16.67% (*n* = 4). After the second dose of vaccination, the most common were local pain 45.83% (*n* = 11), fever 20.83% (*n* = 5), and fatigue 16.67% (*n* = 4). The third dose of vaccination has a similar impact on the PwMS IFN group except for lower incidents of local pain, 29.17% (*n* = 7). Data obtained from the control group revealed that after the first dose, the most frequent adverse events were local pain 21.42% (*n* = 6), fever and headache 17.86% (*n* = 5), as well as muscle pain 10.71% (*n* = 3). After the second dose of vaccination, participants complained about local pain and headache 14.29% (*n* = 4) as well as fever 7.14% (*n* = 2). Mild symptoms with short duration (approximately 1–2 days) were noticed in all groups and after all doses.

## 4. Discussion

Our study examined the immunity status and adverse events occurring after SARS-CoV-2 vaccination in a subgroup of individuals with multiple sclerosis who had previously contracted SARS-CoV-2 and were being treated with dimethyl fumarate and interferon beta. When vaccines aimed at preventing severe cases of COVID-19 were developed, their effects on this specific group of patients, especially those treated with immunosuppressive and immunomodulating drugs, were largely unknown. Over time, initial scientific reports indicated that vaccinations against SARS-CoV-2 are generally safe for PwMS, even for those undergoing disease-modifying therapy (DMT) [45,46]. However, there have also been reports suggesting that several DMTs may reduce humoral immunity (e.g., ocrelizumab, fingolimod, siponimod) [47,48,49,50] and cellular immunity following vaccination (e.g., ocrelizumab, cladribine, interferon beta, fingolimod) [51,52,53]. In the case of anti-CD20 drugs, it is suggested that it may be an effect of B-cell depletion, which involves lower B-cell activity and lower humoral and cellular immunity levels [47]. The decrease of involved humoral and cellular immunity in PwMS treated with sphingosine-1-phosphate receptor modulator (S1P, e.g., siponimod, fingolimod) is probably caused by lower expression of S1P receptors on lymphocytes and reduce their count, what may decrease obtained humoral and cellular immunity levels [50]. Cladribine may have an influence on decreased cellular immunity levels, in fact, causing reconstitution of immunity [53]. However, it is important to emphasize that the data on one of the most frequently used disease-modifying therapies (DMTs) [33,34], such as dimethyl fumarate and interferon beta, are limited. Furthermore, it has been observed that adverse events following vaccination are mostly mild and have a short duration, similar to those in the general population. This observation aligns with the findings of Czarnowska et al. and Ciampi et al., although there is a discrepancy in the percentage distribution of individual symptoms [45,46].

Taking the above into consideration, we decided to conduct our investigation with groups of PwMS treated with interferon beta and dimethyl fumarate. It was evident that humoral immunity would decrease over time; thus, in addition to analyzing IgG (S) anti-SARS-CoV-2 concentrations, we measured interferon gamma titers as a marker of cellular immunity against SARS-CoV-2. This approach complements humoral immunity and may help maintain a protective role against severe outcomes of COVID-19 [43,44]. In our investigation, we selected two populations of PwMS treated with dimethyl fumarate and interferon beta, as well as a group of healthy individuals as a control group. Every effort was made to select participants who corresponded to the typical profile of patients who have multiple sclerosis (described in the Methods section). Individuals from both the study and control groups were vaccinated against SARS-CoV-2, largely receiving two doses of BNT162b2.

In previous studies, seroconversion has been documented following both SARS-CoV-2 infection and vaccination against the virus. Bsteh et al. reported that 76% of patients with multiple sclerosis (PwMS) who had undergone SARS-CoV-2 infection, confirmed via RT-PCR testing approximately 5.2 months post-infection, exhibited detectable levels of IgG (S) antibodies against SARS-CoV-2. Furthermore, this study revealed that PwMS receiving immunomodulatory treatment were more likely to develop antibodies compared to those treated with immunosuppressive agents, although both groups demonstrated lower seroconversion rates than the general population [54]. In alignment with these findings, Sormani et al. examined a cohort of 423 patients with confirmed positive RT-PCR results or symptomatic COVID-19 and found that 76% exhibited positive IgG anti-SARS-CoV-2 levels, with a slightly lower rate of 73.5% observed among PwMS with solely positive RT-PCR results. Blood samples were collected at a median of 75 days following symptom onset. Notably, Sormani et al. also reported a decline in anti-SARS-CoV-2 IgG titers over a 90-day period post-infection, a finding that may aid in determining whether seroconversion among our study participants occurred as a result of SARS-CoV-2 infection or vaccination [55]. In another investigation involving PwMS with confirmed SARS-CoV-2 infection (*n* = 187) and clinically suspected COVID-19 (*n* = 56) but who had not been vaccinated, it was found that 83.44% exhibited positive IgG antibody results. This study indicated that antibodies could be detected up to 13.1 months following COVID-19 diagnosis, which contrasts with the findings of Sormani et al. Additionally, the proportion of anti-SARS-CoV-2 IgG (N) antibodies was found to be higher than that of anti-SARS-CoV-2 IgG (S) antibodies, particularly among PwMS treated with interferon [56], which could be related to the type of seroconversion. Ciampi et al. reported that 66.9% of a cohort of 178 PwMS displayed positive titers of anti-SARS-CoV-2 S1 antibodies four weeks after vaccination against SARS-CoV-2, including six PwMS who presented with clinical symptoms. Notably, a larger cohort of PwMS received an inactivated vaccine, while those vaccinated with an mRNA vaccine demonstrated a higher positivity rate of 78.4% than 62.6% reached after usage of inactivated SARS-CoV-2 vaccines. A higher percentage of positive anti-SARS-CoV-2 S1 antibodies were also reported in PwMS treated with antiCD-20 DMTs. The results are in line with the investigation performed on a larger cohort in the general population. Interestingly, nearly 100% of positive antibody results were observed in PwMS treated with immunomodulatory disease-modifying therapies (DMT) [46,57]. Sormani et al. reported that after 4 weeks after mRNA anti-SARS-CoV-2 vaccination, PwMS mRNA-1273 had greater antibody titers than BNT162b2 [47]. There were no significant variations in seroconversion rates between groups vaccinated with vector and mRNA anti-SARS-CoV-2 vaccines in healthy controls and PwMS, with the exception of a higher percentage of seroconversion in PwMS treated with immunosuppressive DMTs when vector vaccines were administered [58]. Taking the previous findings into account, mRNA vaccines were a better alternative than vector or inactivated vaccines in the majority of PwMS cases.

As we reported, seroconversion approached nearly 100% in both study groups, aligning with findings from Bsteh et al., who collected blood samples three months after a single vaccine dose, and Sormani et al., who measured IgG (S) anti-SARS-CoV-2 levels two weeks post-second dose of vaccination [47,58]. Milo et al. reported a slightly lower seroconversion rate of 75% following the second and third doses of the anti-SARS-CoV-2 vaccination (BNT162b2). Notably, among patients with multiple sclerosis (PwMS) treated with ocrelizumab and fingolimod, a significant proportion (218 out of 522 individuals) exhibited a decrease in the levels of anti-IgG antibodies against SARS-CoV-2. This observation suggests a potential impact of these treatments on the immune response to vaccination [59].

This indicates that the seropositive status of PwMS vaccinated against SARS-CoV-2 remains stable over time. Initial results showed no statistical significance in mean titers of IgG (S) anti-SARS-CoV-2 antibodies; hence, we decided to categorize our patients’ blood sample analysis into two groups: those who obtained samples less than 200 days and more than 200 days after their last vaccination. Our findings revealed that during the first 200 days post-vaccination, the humoral immunity levels in our PwMS subgroups were relatively similar to those of the control group. These results are consistent with those of Sormani et al., Lambrianides et al., who measured serum antibodies three months post-vaccination, and Krajnc et al., who reported results five months after the second dose [47,60,61]. Unexpectedly, over 200 days post-vaccination, both PwMS subgroups exhibited significantly lower mean concentrations of IgG (S) anti-SARS-CoV-2 antibodies compared to the control group.

Investigating the features concerning PwMS treated with dimethyl fumarate (DMF) or interferon beta (IFN), particularly in relation to the estimated concentration of IgG (S) anti-SARS-CoV-2 antibodies, is crucial. Available data presented by Milo et al. described PwMS treated with DMF and IFN six months after their last SARS-CoV-2 vaccination. The findings revealed that the mean concentration of IgG (S) anti-SARS-CoV-2 antibodies was slightly lower in both groups of PwMS than in the control group (*p* < 0.001), which aligns with our results [59]. In contrast, Maglione et al. conducted a similar study but did not find any significant differences in IgG (S) concentrations between PwMS treated with DMF or IFN and the control group [62]. This suggests that PwMS may have an appropriate immune response, but their antibody levels decline more rapidly than those in the control group, indicating that booster vaccinations may be beneficial. To draw definitive conclusions, further investigations with larger sample sizes are necessary.

Furthermore, data on the concentration of IgG (N) anti-SARS-CoV-2 antibodies were collected. It was unexpected that a number of PwMS in the study group (DMF 50%, IFN 66.67%) and the control group (57.14%) exhibited low levels of antibodies. This finding was attributed to a small percentage of participants who had previously undergone SARS-CoV-2 infection across all investigated groups. The IgG (N) anti-SARS-CoV-2 antibodies displayed similar concentrations, with no statistically significant differences, and should not interfere with the accurate assessment of humoral immunity following vaccination. However, data on SARS-CoV-2 infection in our cohorts were restricted, making it difficult to identify the source of obtained humoral anti-SARS-CoV-2 immunity.

What is more, we also tested cellular immunity after vaccination against SARS-CoV-2 based on interferon-gamma secretion by activated T lymphocytes [63]. Our study shows that the mean concentration of interferon-gamma in PwMS subgroups is significantly lower than in the control group. In contrast, data obtained by Trümpelmann et al. show that PwMS treated with IFN beta have cellular immunity levels comparable to those of the general population. Although there were some differences, Trümpelmann et al. conducted their research with samples taken six weeks after the second dose of vaccination, and most of the PwMS were vaccinated with the mRNA-1273 vaccine [64]. A similar observation was made by Maglione et al., who discovered that one and six months after immunization against SARS-CoV-2, the concentration of interferon-gamma was comparable in groups of patients with MS treated with dimethyl fumarate (DMF), IFN beta, and the general population [62]. Additionally, an interesting finding by Tortorella et al. indicated that PwMS treated with IFN beta had significantly lower interferon gamma titers than those measured in healthcare workers (blood collection was performed 2–4 weeks after vaccination) [51]. To verify this thesis, we decided to divide the groups similarly to our analysis of IgG (S) antibodies. In the category with a time duration between vaccination and blood sample collection of fewer than 200 days, the interferon-gamma titers in the study subgroups were lower than those in the controls, but statistical significance was absent. However, an assessment of samples collected from groups more than 200 days after vaccination against SARS-CoV-2 revealed that mean interferon gamma titers in the study subgroups were significantly lower than in the control group. The obtained results may suggest that PwMS have fewer activated T lymphocytes or that interferon gamma has a shorter duration of activity.

## 5. Conclusions

To sum up, our study shows that humoral immunity following vaccination against SARS-CoV-2 in PwMS treated with dimethyl fumarate (DMF) and interferon beta (IFN) is adequate during the first 200 days, comparable to that of the general population. After this period, immunity decreases and is lower than in individuals without multiple sclerosis, although it remains present. Furthermore, individuals with multiple sclerosis receiving DMF or IFN beta treatment exhibited a similar SARS-CoV-2 infection rate and comparable concentrations of IgG (N) anti-SARS-CoV-2 antibodies relative to the general population. Importantly, PwMS treated with DMF and IFN beta had lower concentrations of interferon-gamma compared to the general population. Protective titers of antibodies and interferon-gamma against severe COVID-19 outcomes have not been established [65]. This finding may suggest an earlier loss of post-vaccination immunity in PwMS treated with selected disease-modifying therapies (DMTs). Our investigation has several limitations, including a small sample size and the specific markers measured. Additionally, the lack of data concerning relapses, steroid usage, and SARS-CoV-2 infection in the study group was due to incomplete information in the surveys. In addition, due to the high percentage of mRNA vaccinations in our cohorts, no investigation of various forms of SARS-CoV-2 vaccines was conducted. Moreover, the surveys were conducted simultaneously with blood collection during a specific time period post-vaccination, which may have decreased the accuracy of the collected data. Therefore, further studies and follow-ups are necessary.

## Figures and Tables

**Table 1:** Characteristics of the study and control groups.

Group	Count	Mean Age	Female	Male
Study group (DMF + IFN)	72	43.14 ± 2.79 (95%) ^1^	72.22%(*n* = 52)	27.78% (*n* = 20)
DMF	48 (66.67%)	40.4 ± 3.13 (95%) ^1^	70.83% (*n* = 34)	29.19% (*n* = 14)
IFN	24 (33.33%)	48.63 ± 4.88 (95%) ^1^	75% (*n* = 18)	25% (*n* = 6)
Control Group	28	44.57 ± 4.3 (95%) ^1^	85.71% (*n* = 24)	14.29% (*n* = 4)

^1^ Mean (Confidence Interval); DMF—dimethyl fumarate; IFN—interferon beta.

**Table 2:** Time from last vaccination to blood sample collection in the study and control groups.

Group	Time from Last Vaccination to Blood Sample Collection (Days)
Study group (DMF + IFN)	320.54 (321; 180.5; 436.5) ^1^
DMF	320.56 (282; 180.5; 466.5) ^1^
IFN	320.5 (344.5; 197.5; 385.5) ^1^
Control Group	316.29 (262; 184; 440) ^1^

^1^ Mean (Median; Q1; Q3); DMF—dimethyl fumarate; IFN—interferon beta.

**Table 3:** Detailed characteristics of the studied groups’ vaccination status.

Vaccination		Study Group		Control Group (*n* = 28)
		DMF (*n* = 48)	IFN (*n* = 24)	
1st dose	BNT162b2	79.17% (*n* = 38)	79.17% (*n* = 19)	96.43% (*n* = 27)
	mRNA-1273	12.5% (*n* = 6)	20.83% (*n* = 5)	3.57% (*n* = 1)
	Ad26.COV2.S	4.17% (*n* = 2)	-	-
	NVX-CoV2373	2.08% (*n* = 1)	-	-
	ChAdOx1 nCoV-19	2.08% (*n* = 1)	-	-
2nd dose	BNT162b2	79.17% (*n* = 38)	79.17% (*n* = 19)	96.43% (*n* = 27)
	mRNA-1273	12.5% (*n* = 6)	20.83% (*n* = 5)	3.57% (*n* = 1)
	NVX-CoV2373	2.08% (*n* = 1)	-	-
	ChAdOx1 nCoV-19	2.08% (*n* = 1)	-	-
3rd dose	BNT162b2	60.42% (*n* = 29)	58.33% (*n* = 14)	74.07 % (*n* = 20)
	mRNA-1273	-	3.6% (*n* = 1)	3.57% (*n* = 1)

**Table 4:** Mean and median concentrations of antibodies IgG (S) anti-SARS-CoV-2 in the study and control group divided into <200 and >200 days after the last dose of SARS-CoV-2 vaccination.

Group	Time from Last Vaccination to Sample Collection (Days)	Number of Patients	Mean [AU/mL]	Median [AU/mL]	*p*-Value ^1^
DMF	<200	15	29,840.93	22,624.6 (6525.9; 37,955.5) ^2^	*p* = 0.0013
	>200	33	8990.37	3886.9 (1224.5; 12,550.25) ^2^	
IFN	<200	6	61,966.05	29,769.2 (12,005.2; 76,249.6) ^2^	*p* = 0.0027
	>200	18	9278.12	3203.8 (981.7; 10,146.2) ^2^	
Control	<200	9	32,029.68	27,298 (8043.6; 62,862.6) ^2^	*p* = 0.2281
	>200	19	16,319.34	9544.9 (3145.7; 31,571.1) ^2^	

^1^ Kruskal–Wallis Test, ^2^ Median (Q1, Q3); DMF—dimethyl fumarate, IFN—interferon beta.

**Table 5:** Characteristics of IgG (N) anti-SARS-CoV-2 in the study and control groups.

Groups Studied	Percentage of Negative Results	Mean	Median
DMF	50% (*n* = 24)	2.46	1.1 (0.13; 4.0)
IFN	66.67% (*n* = 16)	2.17	0.64 (0.1; 2.88)
DMF + IFN	55.56% (*n* = 40)	2.36	0.87 (0.12; 3.68)
Control	57.14% (*n* = 16)	1.82	0.74 (0.17; 2.74)

DMF—dimethyl fumarate; IFN—interferon beta.

## Data Availability

Data are contained within the article and Supplementary Materials.

## Supplementary Materials

The following supporting information can be downloaded at: https://www.mdpi.com/article/10.3390/biomedicines13010153/s1, Figure S1: Patient Recruitment Flowchart.

## References

[1] Adil M.T., Rahman R., Whitelaw D., Jain V., Al-Taan O., Rashid F., Munasinghe A., Jambulingam P. (2021). SARS-CoV-2 and the Pandemic of COVID-19. Postgrad. Med. J..

[2] CDC Museum COVID-19 Timeline. https://www.cdc.gov/museum/timeline/covid19.html.

[3] Coronavirus Tracker. https://www.worldometers.info/coronavirus/.

[4] Sarker R., Roknuzzaman A.S.M., Nazmunnahar, Shahriar M., Hossain M.J., Islam M.R. (2023). The WHO has Declared the End of Pandemic Phase of COVID-19: Way to Come Back in the Normal Life. Health Sci. Rep..

[5] da Rosa Mesquita R., Francelino Silva L.C., Santos Santana F.M., Farias de Oliveira T., Campos Alcântara R., Monteiro Arnozo G., Rodrigues da Silva Filho E., Galdino Dos Santos A.G., Oliveira da Cunha E.J., Salgueiro de Aquino S.H. (2021). Clinical Manifestations of COVID-19 in the General Population: Systematic Review. Wien. Klin. Wochenschr..

[6] Jafari-Oori M., Ghasemifard F., Ebadi A., Karimi L., Rahimi-Bashar F., Jamialahmadi T., Guest P.C., Vahedian-Azimi A., Sahebkar A. (2021). Acute Respiratory Distress Syndrome and COVID-19: A Scoping Review and Meta-Analysis. Adv. Exp. Med. Biol..

[7] Wan Y., Shang J., Graham R., Baric R.S., Li F. (2020). Receptor Recognition by Novel Coronavirus from Wuhan: An Analysis Based on Decade-Long Structural Studies of SARS Coronavirus. J. Virol..

[8] Gudowska-Sawczuk M., Mroczko B. (2021). The Role of Neuropilin-1 (NRP-1) in SARS-CoV-2 Infection: Review. J. Clin. Med..

[9] Fu Y., Pan Y., Li Z., Li Y. (2021). The Utility of Specific Antibodies Against SARS-CoV-2 in Laboratory Diagnosis. Front. Microbiol..

[10] Treggiari D., Piubelli C., Caldrer S., Mistretta M., Ragusa A., Orza P., Pajola B., Piccoli D., Conti A., Lorenzi C. (2022). SARS-CoV-2 Rapid Antigen Test in Comparison to RT-PCR Targeting Different Genes: A Real-Life Evaluation Among Unselected Patients in a Regional Hospital of Italy. J. Med. Virol..

[11] Ghaffari A., Meurant R., Ardakani A. (2020). COVID-19 Serological Tests: How Well Do They Actually Perform?. Diagnostics.

[12] Gote V., Bolla P.K., Kommineni N., Butreddy A., Nukala P.K., Palakurthi S.S., Khan W. (2023). A Comprehensive Review of mRNA Vaccines. Int. J. Mol. Sci..

[13] Rydyznski Moderbacher C., Kim C.J., Mateus J., Plested J.S., Zhu M., Cloney-Clark S., Weiskopf D., Sette A., Fries L., Glenn G. (2022). NVX-CoV2373 Vaccination Induces Functional SARS-CoV-2-Specific CD4^+^ and CD8^+^ T Cell Responses. J. Clin. Investig..

[14] Vanaparthy R., Mohan G., Vasireddy D., Atluri P. (2021). Review of COVID-19 Viral Vector-Based Vaccines and COVID-19 Variants. Infez. Med..

[15] Padoan A., Sciacovelli L., Basso D., Negrini D., Zuin S., Cosma C., Faggian D., Matricardi P., Plebani M. (2020). IgA-Ab Response to Spike Glycoprotein of SARS-CoV-2 in Patients with COVID-19: A Longitudinal Study. Clin. Chim. Acta.

[16] Assaid N., Arich S., Charoute H., Akarid K., Anouar Sadat M., Maaroufi A., Ezzikouri S., Sarih M. (2022). Kinetics of SARS-CoV-2 IgM and IgG Antibodies 3 Months After COVID-19 Onset in Moroccan Patients. Am. J. Trop. Med. Hyg..

[17] Liu A., Wang W., Zhao X., Zhou X., Yang D., Lu M., Lv Y. (2020). Disappearance of Antibodies to SARS-CoV-2 in a -COVID-19 Patient after Recovery. Clin. Microbiol. Infect..

[18] Yousefi Z., Taheri N., Dargahi M., Chaman R., Binesh E., Emamian M.H., Jafari R. (2022). Long-Term Persistence of Anti-SARS-COV-2 IgG Antibodies. Curr. Microbiol..

[19] Burbelo P.D., Riedo F.X., Morishima C., Rawlings S., Smith D., Das S., Strich J.R., Chertow D.S., Davey R.T., Cohen J.I. (2020). Sensitivity in Detection of Antibodies to Nucleocapsid and Spike Proteins of Severe Acute Respiratory Syndrome Coronavirus 2 in Patients with Coronavirus Disease 2019. J. Infect. Dis..

[20] Azkur A.K., Akdis M., Azkur D., Sokolowska M., van de Veen W., Bruggen M.C., O’Mahony L., Gao Y., Nadeau K., Akdis C.A. (2020). Immune Response to SARS-CoV-2 and Mechanisms of Immunopathological Changes in COVID-19. Allergy.

[21] Grifoni A., Weiskopf D., Ramirez S.I., Mateus J., Dan J.M., Moderbacher C.R., Rawlings S.A., Sutherland A., Premkumar L., Jadi R.S. (2020). Targets of T Cell Responses to SARS-CoV-2 Coronavirus in Humans with COVID-19 Disease and Unexposed Individuals.

[22] Schwarzkopf S., Krawczyk A., Knop D., Klump H., Heinold A., Heinemann F.M., Thummler L., Temme C., Breyer M., Witzke O. (2021). Cellular Immunity in COVID-19 Convalescents with PCR-Confirmed Infection but with Undetectable SARS-CoV-2-Specific IgG. Emerg. Infect. Dis..

[23] Xu K., Dai L., Gao G.F. (2021). Humoral and Cellular Immunity and the Safety of COVID-19 Vaccines: A Summary of Data Published by 21 May 2021. Int. Immunol..

[24] The Lancet Neurology (2021). Multiple Sclerosis Under the Spotlight. Lancet Neurol..

[25] Potemkowski A. (2009). Multiple Sclerosis in Poland and Worldwide–Epidemiological Considerations. Aktualn. Neurol..

[26] Torkildsen Ø., Myhr K.M., Bø L. (2016). Disease-Modifying Treatments for Multiple Sclerosis—A Review of Approved Medications. Eur. J. Neurol..

[27] Wingerchuk D.M., Carter J.L. (2014). Multiple sclerosis: Current and Emerging Disease-Modifying Therapies and Treatment Strategies. Mayo Clin. Proc..

[28] Pandit L. (2019). No Evidence of Disease Activity (NEDA) in Multiple Sclerosis—Shifting the Goal Posts. Ann. Indian. Acad. Neurol..

[29] Casanova B., Quintanilla-Bordás C., Gascón F. (2022). Escalation vs. Early Intense Therapy in Multiple Sclerosis. J. Pers. Med..

[30] Dello Russo C., Scott K.A., Pirmohamed M. (2021). Dimethyl Fumarate Induced Lymphopenia in Multiple Sclerosis: A Review of the Literature. Pharmacol. Ther..

[31] Achiron A., Mandel M., Dreyer-Alster S., Harari G., Dolev M., Menascu S., Magalashvili D., Flechter S., Givon U., Guber D. (2021). Humoral Immune Response in Multiple Sclerosis Patients Following PfizerBNT162b2 COVID19 Vaccination: Up to 6 Months Cross-Sectional Study. J. Neuroimmunol..

[32] Xavier A., Campagna M.P., Maltby V.E., Kilpatrick T., Taylor B.V., Butzkueven H., Ponsonby A.L., Scott R.J., Jokubaitis V.G., Lea R.A. (2023). Interferon Beta Treatment is a Potent and Targeted Epigenetic Modifier in Multiple Sclerosis. Front. Immunol..

[33] Henderson M., Horton D.B., Bhise V., Pal G., Bushnell G., Dave C.V. (2023). Initiation Patterns of Disease-Modifying Therapies for Multiple Sclerosis Among US Adults and Children, 2001 Through 2020. JAMA Neurol..

[34] Cohan S.L., Hendin B.A., Reder A.T., Smoot K., Avila R., Mendoza J.P., Weinstock-Guttman B. (2021). Interferons and Multiple Sclerosis: Lessons from 25 Years of Clinical and Real-World Experience with Intramuscular Interferon Beta-1a (Avonex). CNS Drugs.

[35] Thompson A.J., Banwell B.L., Barkhof F., Carroll W.M., Coetzee T., Comi G., Correale J., Fazekas F., Filippi M., Freedman M.S. (2018). Diagnosis of Multiple Sclerosis: 2017 Revisions of the McDonald Criteria. Lancet Neurol..

[36] Kurtzke J.F. (1983). Rating Neurologic Impairment in Multiple Sclerosis: An Expanded Disability Status Scale (EDSS). Neurology.

[37] Thomas S.J., Moreira E.D., Kitchin N., Absalon J., Gurtman A., Lockhart S., Perez J.L., Pérez Marc G., Polack F.P., Zerbini C. (2021). Safety and Efficacy of the BNT162b2 mRNA COVID-19 Vaccine Through 6 Months. N. Engl. J. Med..

[38] Levin E.G., Lustig Y., Cohen C., Fluss R., Indenbaum V., Amit S., Doolman R., Asraf K., Mendelson E., Ziv A. (2021). Waning Immune Humoral Response to BNT162b2 COVID-19 Vaccine over 6 Months. N. Engl. J. Med..

[39] Feikin D.R., Higdon M.M., Abu-Raddad L.J., Andrews N., Araos R., Goldberg Y., Groome M.J., Huppert A.H., O‘Brien K.L., Smith P.G. (2022). Duration of Effectiveness of Vaccines Against SARS-CoV-2 Infection and COVID-19 Disease: Results of a Systematic Review and Meta-Regression. Lancet.

[40] Collier A.Y., Yu J., McMahan K., Liu J., Chandrashekar A., Maron J.S., Atyeo C.A., Martinez D.R., Ansel J.L., Aguayo R. (2021). Differential Kinetics of Immune Responses Elicited by COVID-19 Vaccines. N. Engl. J. Med..

[41] Ramos A., Martins S., Marinho A.S., Norton P., Cardoso M.J., Guimarães J.T. (2024). Evaluation of SARS-CoV-2 Interferon Gamma Release Assay in BNT162b2 Vaccinated Healthcare Workers. PLoS ONE..

[42] Goel R.R., Painter M.M., Apostolidis S.A., Mathew D., Meng W., Rosenfeld A.M., Lundgreen K.A., Reynaldi A., Khoury D.S., Pattekar A. (2021). mRNA Vaccines Induce Durable Immune Memory to SARS-CoV-2 and Variants of Concern. Science.

[43] Chivu-Economescu M., Bleotu C., Grancea C., Chiriac D., Botezatu A., Iancu I.V., Pitica I., Necula L.G., Neagu A., Matei L. (2022). Kinetics and Persistence of Cellular and Humoral Immune Responses to SARS-CoV-2 Vaccine in Healthcare Workers With or Without Prior COVID-19. J. Cell. Mol. Med..

[44] Gombolay G.Y., Dutt M., Tyor W. (2022). Immune Responses to SARS-CoV-2 Vaccination in Multiple Sclerosis: A Systematic Review/Meta-Analysis. Ann. Clin. Transl. Neurol..

[45] Czarnowska A., Tarasiuk J., Zajkowska O., Wnuk M., Marona M., Nowak K., Słowik A., Jamroz-Wiśniewska A., Rejdak K., Lech B. (2022). Safety of Vaccines Against SARS-CoV-2 Among Polish Patients with Multiple Sclerosis Treated with Disease-Modifying Therapies. Vaccines.

[46] Ciampi E., Uribe-San-Martin R., Soler B., García L., Guzman J., Pelayo C., Jürgensen L., Guzman I., Vera F., Galleguillos L. (2022). Safety and Humoral Response Rate of Inactivated and mRNA Vaccines Against SARS-CoV-2 in Patients with Multiple Sclerosis. Mult. Scler. Relat. Disord..

[47] Sormani M.P., Inglese M., Schiavetti I., Carmisciano L., Laroni A., Lapucci C., Da Rin G., Serrati C., Gandoglia I., Tassinari T. (2021). Effect of SARS-CoV-2 mRNA Vaccination in MS Patients Treated with Disease Modifying Therapies. EBioMedicine.

[48] Tallantyre E.C., Vickaryous N., Anderson V., Asardag A.N., Baker D., Bestwick J., Bramhall K., Chance R., Evangelou N., George K. (2022). COVID-19 Vaccine Response in People with Multiple Sclerosis. Ann. Neurol..

[49] Bigaut K., Kremer L., Fleury M., Lanotte L., Collongues N., de Seze J. (2021). Impact of Disease-Modifying Treatments on Humoral Response After COVID-19 Vaccination: A Mirror of the Response After SARS-CoV-2 Infection. Rev. Neurol..

[50] Krbot Skorić M., Rogić D., Lapić I., Šegulja D., Habek M. (2022). Humoral Immune Response to COVID-19 Vaccines in People with Secondary Progressive Multiple Sclerosis Treated with Siponimod. Mult. Scler. Relat. Disord..

[51] Tortorella C., Aiello A., Gasperini C., Agrati C., Castilletti C., Ruggieri S., Meschi S., Matusali G., Colavita F., Farroni C. (2022). Humoral- and T-Cell-Specific Immune Responses to SARS-CoV-2 mRNA Vaccination in Patients with MS Using Different Disease-Modifying Therapies. Neurology.

[52] Sainz de la Maza S., Walo-Delgado P.E., Rodríguez-Domínguez M., Monreal E., Rodero-Romero A., Chico-García J.L., Pariente R., Rodríguez-Jorge F., Ballester-González R., Villarrubia N. (2023). Short- and Long-Term Humoral and Cellular Immune Responses to SARS-CoV-2 Vaccination in Patients with Multiple Sclerosis Treated with Disease-Modifying Therapies. Vaccines.

[53] Etemadifar M., Nouri H., Pitzalis M., Idda M.L., Salari M., Baratian M., Mahdavi S., Abhari A.P., Sedaghat N. (2022). Multiple Sclerosis Disease-Modifying Therapies and COVID-19 Vaccines: A Practical Review and Meta-Analysis. J. Neurol. Neurosurg. Psychiatry.

[54] Bsteh G., Dürauer S., Assar H., Hegen H., Heschl B., Leutmezer F., Pauli F.D., Gradl C., Traxler G., Zulehner G. (2021). Humoral Immune Response After COVID-19 in Multiple Sclerosis: A Nation-Wide Austrian Study. Mult. Scler..

[55] Sormani M.P., Schiavetti I., Landi D., Carmisciano L., De Rossi N., Cordioli C., Moiola L., Radaelli M., Immovilli P., Capobianco M. (2022). SARS-CoV-2 Serology After COVID-19 in Multiple Sclerosis: An International Cohort Study. Mult. Scler. J..

[56] Zabalza A., Arrambide G., Tagliani P., Cárdenas-Robledo S., Otero-Romero S., Esperalba J., Fernandez-Naval C., Trocoli Campuzano J., Martínez Gallo M., Castillo M. (2022). Humoral and Cellular Responses to SARS-CoV-2 in Convalescent COVID-19 Patients with Multiple Sclerosis. Neurol. Neuroimmunol. Neuroinflamm..

[57] Sauré D., O’Ryan M., Torres J.P., Zuniga M., Santelices E., Basso L.J. (2022). Dynamic IgG Seropositivity After Rollout of CoronaVac and BNT162b2 COVID-19 Vaccines in Chile: A Sentinel Surveillance Study. Lancet Infect. Dis..

[58] Bsteh G., Hegen H., Traxler G., Krajnc N., Leutmezer F., Di Pauli F., Kornek B., Rommer P., Zulehner G., Dürauer S. (2022). Comparing Humoral Immune Response to SARS-CoV2 Vaccines in People with Multiple Sclerosis and Healthy Controls: An Austrian Prospective Multicenter Cohort Study. Eur. J. Neurol..

[59] Milo R., Staun-Ram E., Karussis D., Karni A., Hellmann M.A., Bar-Haim E., Miller A., Israeli Neuroimmunology Study Group on COVID-19 Vaccination in Multiple Sclerosis (2022). Humoral and Cellular Immune Responses to SARS-CoV-2 mRNA Vaccination in Patients with Multiple Sclerosis: An Israeli Multi-Center Experience Following 3 Vaccine Doses. Front. Immunol..

[60] Lambrianides A., Deeba E., Hadjiagapiou M., Pantzaris M., Krashias G., Christodoulou C. (2023). SARS-CoV-2-Specific Antibody Responses Following BNT162b2 Vaccination in Individuals with Multiple Sclerosis Receiving Different Disease-Modifying Treatments. Front. Neurol..

[61] Krajnc N., Hegen H., Traxler G., Leutmezer F., Di Pauli F., Kornek B., Rommer P., Zulehner G., Riedl K., Dürauer S. (2022). Humoral Immune Response to SARS-CoV-2 Third Vaccination in Patients with Multiple Sclerosis and Healthy Controls: A Prospective Multicenter Study. Mult. Scler. Relat. Disord..

[62] Maglione A., Francese R., Arduino I., Rosso R., Matta M., Rolla S., Lembo D., Clerico M. (2023). Long-Lasting Neutralizing Antibodies and T Cell Response After the Third Dose of mRNA anti-SARS-CoV-2 Vaccine in Multiple Sclerosis. Front. Immunol..

[63] Adu-Berchie K., Obuseh F.O., Mooney D.J. (2023). T Cell Development and Function. Rejuvenation Res..

[64] Trümpelmann S., Schulte-Mecklenbeck A., Steinberg O.V., Wirth T., Fobker M., Lohmann L., Lünemann J.D., Wiendl H., Gross C.C., Klotz L. (2022). Impact of Disease-Modifying Therapies on Humoral and Cellular Immune-Responses Following SARS-CoV-2 Vaccination in MS Patients. Clin. Transl. Sci..

[65] Giossi R., Consonni A., Torri Clerici V., Zito A., Rigoni E., Antozzi C., Brambilla L., Crisafulli S.G., Bellino A., Frangiamore R. (2022). Anti-Spike IgG in Multiple Sclerosis Patients After BNT162b2 Vaccine: An Exploratory Case-Control Study in Italy. Mult. Scler. Relat. Disord..

