# Supplementary figures and images for: Humoral and Cellular Immunity After Vaccination Against SARS-CoV-2 in Relapsing-Remitting Multiple Sclerosis Patients Treated with Interferon Beta and Dimethyl Fumarate
Source: Biomedicines. 2025 Jan 9;13(1):153. doi: 10.3390/biomedicines13010153 (PMC11763107; doi:10.3390/biomedicines13010153)

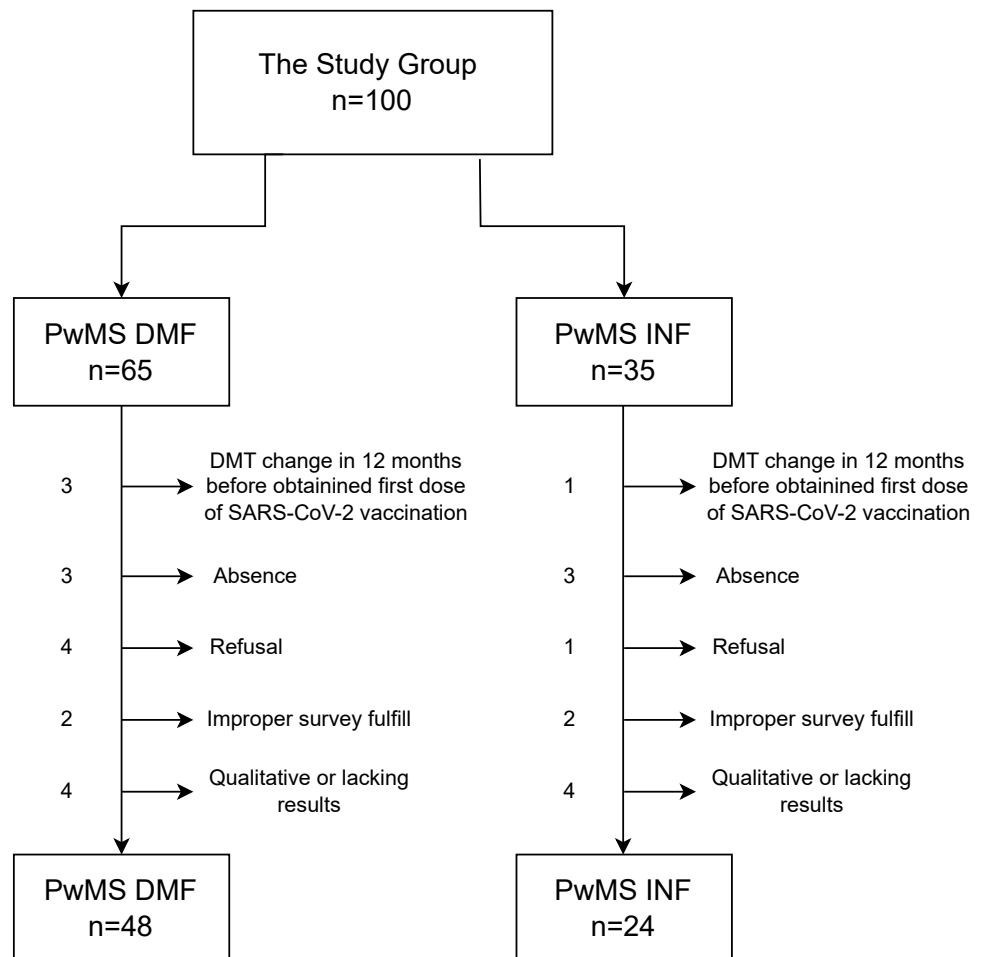

Supplement: Supplementary file 1 [file biomedicines-13-00153-s001.zip › biomedicines-3413946-supplementary.pdf]
